# Rehabilitation Engineering Research Center on Mobile Rehabilitation: State of the Science Conference Report—Future Directions for mRehab for People with Disabilities

**DOI:** 10.3390/ijerph22040532

**Published:** 2025-03-31

**Authors:** John Morris, Mike Jones, Frank DeRuyter, Amanda Rabinowitz, David J. Reinkensmeyer

**Affiliations:** 1 Virginia C. Crawford Research Institute, Shepherd Center, Atlanta, GA 30309, USA; mike.jones@shepherd.org (M.J.); frank.deruyter@duke.edu (F.D.); 2 Jefferson Moss Rehabilitation Research Institute, Elkins Park, PA 19027, USA; amanda.rabinowitz@jefferson.edu; 3 Henry Samueli School of Engineering, University of California, Irvine, CA 92697, USA; dreinken@uci.edu

**Keywords:** mobile health, mHealth, mRehab, disability, rehabilitation, information and communication technology, accessibility, community participation, health and function

## Abstract

This article summarizes proceedings of the State of the Science (SOS) Conference on Information and Communication Technology (ICT) Access for Mobile Rehabilitation, convened by the Rehabilitation Engineering Research Center on Mobile Rehabilitation (mRehab RERC), which is funded by the U.S. National Institute on Disability, Independent Living and Rehabilitation Research (NIDILRR). The conference sought to assess the current state of the field and identify future research and development priorities for the field of mobile rehabilitation. The conference comprised four sessions addressing the following broad areas: (1) adherence to and effectiveness of home therapeutic exercise programs (HEPs); (2) technology for remote monitoring to support rehabilitation in the home and community (mRehab); (3) analytic techniques for using “big data” generated by remote monitoring to customize home exercise; and (4) barriers and facilitators to adoption of mRehab technology. Priorities for further research and development were identified using a three-stage process of gathering and refining expert opinions informed by the Delphi method for identifying future states in specific fields of inquiry. Results: Eight research and six technology development priorities were identified in the third and last stage of refinement of the initial set of priorities identified during the SOS Conference.

## 1. Introduction

The Rehabilitation Engineering Research Center on Mobile Rehabilitation (mRehab RERC) is a partnership between Shepherd Center, University of California, Irvine (UCI), Moss Rehabilitation Research Institute (MRRI), and commercial partners Flint Rehab and Pt Pal. The RERC’s mission is to ensure access by people with disabilities to advanced mobile rehabilitation technologies that will improve adherence, engagement, and outcomes of home-based therapeutic interventions (www.mrehabrerc.org). Goals include developing, testing, commercializing, and evaluating the effectiveness of accessible, evidence-based mRehab interventions and technologies that have the potential to maximize the health and function of individuals with some of the most significant disabilities—those with or at risk of developing chronic health conditions. The mRehab RERC State of the Science Conference (SOS Conference) was held in Atlanta in October 2023 as part of the annual meeting of the American Congress for Rehabilitation Medicine (ACRM).

Mobile rehabilitation (mRehab) is closely related to mHealth [1] but focuses on rehabilitation of function versus health and wellness overall. The term has been used to refer to mobile device telerehabilitation [2] and to a smartphone-supported intervention for hand rehabilitation for people with stroke [3]. We define mRehab as the delivery of services and support for rehabilitation in the home and community using mobile information and communication technologies (ICT) [4]. The mRehab RERC primarily addresses rehabilitation for people living with the motor, cognitive and psychosocial effects of injury and other disabling conditions (e.g., brain injury, spinal cord injury, stroke, multiple sclerosis).

Ongoing development and widespread adoption of powerful, mobile ICT has enabled novel and increasingly accessible technology-based health and rehabilitation interventions and support for community-dwelling individuals, including those with disabling chronic conditions.

mRehab can rely on standalone solutions for health self-management by individuals in the community or can be integrated into therapy management systems that support information sharing and communication with clinical professionals. As with mHeatlh, mRehab offers potential for keeping patients engaged in therapy through features like personalization, self-monitoring, goal setting, reminders, and feedback [5,6]. These, in turn, support continued exercise activity and healthy behaviors outside of the clinic and can provide low-cost, accessible tools to support these therapeutic exercises and behaviors. mRehab allows regular tracking (daily, hourly, and at even shorter intervals) of individual behavior, activity, exercise, and general health [7,8].

The potential of these technology-enabled interventions and supports has drawn the attention of researchers, clinicians, engineers, non-governmental organizations, and national policy makers over the past two decades. Dobkin and Dorsch summarized the need for expansion of mHealth/mRehab services in 2011, noting that clinicians who treat patients with chronic conditions need to measure gains and losses of daily functioning over time, assess effects of timing and dose of interventions and modify prescriptions as needed, provide adequate feedback about patient performance, and update instructions more frequently than only during in-clinic visits [7].

More recently, U.S. federal health research agencies and the World Health Organization have emphasized the growing need to conduct research and development activities on technologies to improve health and function and expand access to health and rehabilitation services. The 2017 Plan on Rehabilitation published by the U.S. National Institutes of Health (NIH) outlined the potential of information and communication technologies (ICT) to transform rehabilitation services with mobile technologies [8]. The NIH Plan on Rehabilitation highlighted the utility of ICT in expanding access to rehabilitation, noting that “the use of ICT eliminates distance barriers and can make rehabilitation and healthcare services available to people who have limited access to transportation and other access issues”. In its updated Plan on Rehabilitation (2021), the NIH has continued to emphasize “increase[d] access to rehabilitation services through telehealth and remote assessment, delivery of care, and adherence monitoring. … combining novel sensors and technology with the science of behavior change and motivation research”. The 2021 NIH plan also called for “[a]ugmented intelligence systems to process and interpret data from individuals and populations…. includ[ing] development of intelligent systems for processing multimodal data available from existing and new sensing systems and applying these data to laboratory and community settings” [9]. NIDILRR’s Long-Range Plan 2024–2028 also emphasizes the need for R&D in rehabilitation technologies, including “cloud computing, machine learning, artificial intelligence, pervasive information, big data and analytics, rapid design and fabrication, advanced materials, micro-electro-mechanical systems, sensor technologies, and advanced communication technologies” [10].

These calls to action to expand access to healthcare and rehabilitation services via new rehabilitation technologies are central to the World Health Organization’s Rehabilitation 2030 Initiative, a core principle of which is that “rehabilitation should be available for all the population and through all stages of the life course” [11]. The WHO initiative notes that “efforts to strengthen rehabilitation should be directed toward … integrating rehabilitation into all levels of health care”. Expanding access to rehabilitation services was also highlighted in the WHO’s 2023 declaration on “Strengthening Rehabilitation in Health Systems”, in which member states were urged to develop strategies to “allow rehabilitation to reach underserved rural, remote and hard-to-reach areas” [12].

Government policymakers have also recognized the need for mRehab particularly through the introduction of reimbursement policies to cover remote, technology-enabled interventions. In the United States, the Center for Medicare and Medicaid Services (CMS) has introduced a series of annual updates to its reimbursement regulations for remote physiologic monitoring (RPM; non-invasive medical monitoring remotely) in 2018, treatment in 2019, and remote therapeutic monitoring in 2022 [13].

Recent global demographic and health trends have exacerbated the need for mHealth/mRehab to fill gaps in existing healthcare delivery structures. The COVID-19 global health emergency overwhelmed hospital- and clinic-based care, and social distancing measures disturbed in-person provision of services [14]. Other factors contributing to the need for mRehab include rapidly aging populations [15,16], growing old-age dependency ratios [15], longer life expectancy in general [17] and for people with severe disabilities [18], and the growing shortage of healthcare professionals in the U.S. [19] and worldwide [20]. CMS expanded reimbursement coverage for RPM in response to the COVID-19 public health emergency by permanently clarifying that healthcare providers can utilize RPM billing codes for acute and chronic conditions, allowing reimbursement for monitoring even during the post-acute phase.

Despite the need and opportunity for mHealth/mRehab solutions and systems, challenges to successful development and implementation remain. The 2017 NIH Plan on Rehabilitation noted several barriers to uptake and usability of mRehab, including limited scientific evidence, lack of integration of multiple perspectives and disciplines to ensure proper workflow, data privacy and security, and limited reimbursement. Importantly, the authors note: “Of particular concerns for the disability community is how factors at the human-technology interface can impose barriers to use” [8]. These concerns are echoed in the NIDILRR’s 2024–2028 Long-Range Plan and the WHO Plan for Strengthening Rehabilitation in Healthcare highlights, both of which emphasize the need for access to rehabilitation and user-centered design and development to ensure accessibility, usability, and effectiveness of rehabilitation and assistive technologies [10,12].

Our experience after 4+ years working on mRehab RERC research and development activities suggests additional challenges and needs: (1) inclusive technology development and validation activities that ensure that solutions meet the needs and desires of all users, including marginalized populations (racial/ethnic/linguistic minorities, low-resource and rural populations, rehabilitation clinicians, billing staff, and payers); (2) deeper understanding of mechanisms supporting patient adherence to prescribed home rehabilitation exercises (e.g., gamification, patient access to activity and health data, supportive messaging); (3) deeper understanding of clinician strategies when prescribing home exercise programs (HEPs); (4) expanded insurance reimbursement (by private insurers and national health systems) for mRehab services and simplification of reimbursement policies; (5) common data elements and shared datasets across the many research and development efforts in this area. These challenges and needs are described in greater detail in the Results Section where summaries of each of the 12 presentations are provided.

## 2. Materials and Methods

The 2-day mRehab RERC SOS Conference comprised 4 sessions, each with 3 presentations followed by discussion, organized under the following broad areas of inquiry:

- Session 1: Adherence to and effectiveness of home/remote therapeutic exercise.
- Session 2: Technology for remote monitoring and support.
- Session 3: Analytic techniques for managing “Big Data” available from mRehab.
- Session 4: Barriers and facilitators to uptake and adoption of mRehab.

Conference attendees included mRehab RERC staff, invited speakers, and active audience participants representing clinical, administration, research, engineering, and business startup backgrounds. Each session included at least 2 researchers or engineers from the mRehab RERC. NIDILRR requires that SOS Conferences for the RERC program include substantial coverage of the work produced by each funded center over the previous 4 years of their 5-year grant. Additionally, each session included at least 1 invited speaker from outside the RERC to ensure inclusion of external perspectives.

The presentations addressed key concerns and opportunities for the future of mobile rehabilitation in the home and community, including the following: (1) adherence to prescribed HEPs; (2) economic, social, and cultural relevance of mRehab solutions; (3) patient-centered research and development; (4) gamification combined with sensor technology; (5) artificial intelligence conversational agents (AI chatbots); (6) facilitating uptake/use of wearable mRehab technology; (7) just-in-time adaptive interventions (JITAIs); (8) interpreting acceleration data for measuring participation; (9) exercise habit formation; (10) integrating mRehab technologies into clinical practice; (11) regulatory and reimbursement environment in the U.S.; and (12) implementing mRehab in outpatient clinics in the U.S.

### Methods

The conference organizers utilized a process informed by the Delphi method of consensus-building research to identify a list of priority needs and opportunities for ongoing research and engineering to advance the state of the science in mobile rehabilitation. The Delphi method or technique was originally developed by the RAND Corporation in the 1950s to “obtain the most reliable opinion of a group of experts by subjecting them to a series of questionnaires interspersed with controlled opinion feedback” to model possible future states of a particular issue, problem, or policy area [21].

The Delphi method is characterized by four methodological features:

1. A group of expert panelists is questioned about the issue of interest;
2. Participation is anonymous to promote independent input and avoid social pressure;
3. It is an iterative process comprising several rounds of inquiry, review and response;
4. Each subsequent round is informed by a summary of the group response of the previous round.

Since the 1950s, and especially in the past 15 years, the Delphi technique has been used with increasing frequency to develop consensus-based policies, guidelines for best practice, and exploration of future states on specific issues, including health and medicine [22]. One study published in 2023 shows that approximately 65% of all published studies using the Delphi method were in medical journals, and a third of all published studies came out in the first 3 years of the 2020s [23]. A search on PubMed, the online searchable database of biomedical and life sciences literature maintained by the U.S. National Library of Medicine, shows over 3800 publications on the Delphi method in 2023 and 2024 on a wide range of health topics in numerous countries and geographies, including interprofessional management of patients with dementia in Australia [24]; post-graduate anesthesia training in China [25]; implementation of digital surveillance tools in Malawi [26]; international research priorities of ADHD professionals [27]; assessing the quality of AI chatbot-generated responses in making healthcare decisions in the U.S. [28]; and the development of a training system for infectious disease specialist nurses in China [29]. Delphi studies usually involve 3 or 4 rounds of data collection and item refinement. Initially, experts are provided with a dataset and other information and perspectives to generate a wide range of ideas and estimates of their importance. In subsequent rounds, participants evaluate the importance of individual items and may propose changes to their presentation or substance. As the process advances to each subsequent round of data gathering and item refinement, the number of expert participants is often reduced to encourage more detailed and higher-quality feedback. It is expected that the numerous items and divergent ratings of importance identified in the first round will yield to increasing convergence of estimates through repeated engagement of the panel of experts.

**Figure 1:**
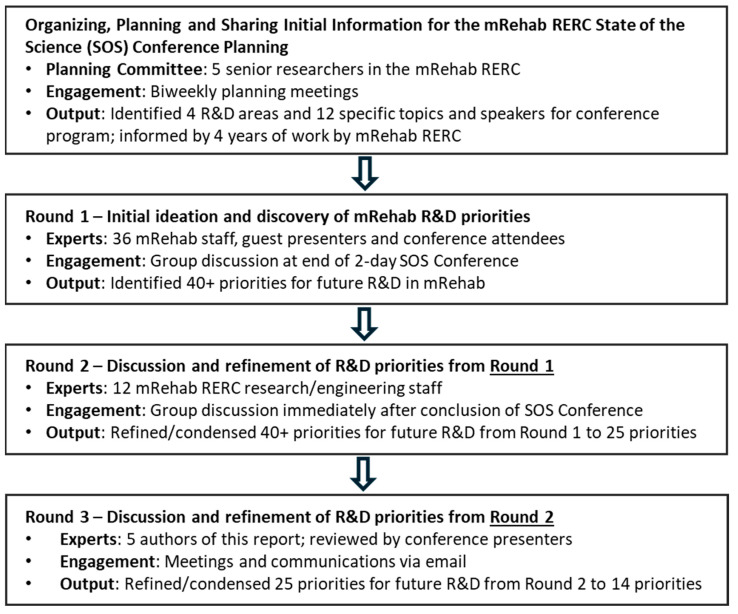
Research and development priorities for mobile rehabilitation: three rounds of expert input and refinement.

The mRehab RERC State of the Science Conference incorporated the general structure and activities of the Delphi method to identify research and development priorities for the field of mobile health and rehabilitation (Figure 1). The design utilized is similar to the Delphi method in several ways:

1. Information sharing. Data and other information and perspectives were provided to all attendees and participants (mRehab RERC staff, speakers, and members of the American Congress of Rehabilitation Medicine who attended the SOS Conference) via thematically organized presentations and related discussion. Additionally, prior to the conference, the organizers provided a program with abstracts of all the presentations. These data and background information were developed by the SOS Conference organizers over the previous 12 months through multiple sessions of ideation and conference planning.
2. Round 1. The initial round of broad discovery of R&D needs and opportunities for mobile rehabilitation was conducted via an interactive ideation session among all staff, speakers and attendees (approximately 36 rehabilitation R&D professionals) in a two-hour session at the end of the SOS Conference. Participants were asked to identify a list of key priorities for the field, informed by the 12 presentations and discussions and by the participants’ own knowledge and experience as researchers, engineers, clinicians and administrators.
3. Round 2. Refinement and interactive discussion of the ideas put forth in Round 1 were conducted among 12 mRehab RERC staff (which included several conference speakers) immediately after the SOS Conference.
4. Round 3. Further refinement of the set of R&D priorities was conducted remotely via videoconferences and email among the 5 authors of this paper over the weeks following the SOS Conference.

Our approach diverged from the traditional Delphi method in several important ways:

1. The selection process for the Round 1 panel of experts among conference attendees was not fully structured. Other than the members of the mRehab RERC and invited speakers, participants were not specifically recruited. Instead, the process was adapted to fit the structure and functioning of the annual conference of a professional society in rehabilitation medicine. We simply engaged all attendees at the annual meeting of the American Congress for Rehabilitation Medicine who independently registered and attended our SOS Conference. Despite the voluntary nature of participation, all participants in Round 1 were research, engineering, administrative and clinical professionals in the area of physical medicine and rehabilitation (PM&R) and rehabilitation technology.
2. The experts in each round were not restricted to anonymous, asynchronous participation. All rounds involved direct interaction among participants. This did not seem to inhibit active participation or impact the nature of participant feedback.
3. Rounds 2 and 3 involved only experts from the mRehab RERC: principal investigators, project directors and research staff from Shepherd Center, Moss Rehabilitation Research Institute, University of California at Irvine, Flint Rehab and Pt Pal.
4. Evaluation and refinement of R&D priorities relied on an iterative process of direct discussion and review, instead of a formal rating system or scale.

## 3. Results

### 3.1. Session 1: Adherence to and Effectiveness of Home/Remote Therapeutic Exercise

The first session of the SOS Conference focused on adherence and effectiveness of home/remote therapeutic exercise and technologies (Table 1). The core conundrum is that completion of prescribed HEPs is believed to contribute to greater functional recovery and patient satisfaction, but adherence to HEPs is low and short-lived [30]. Compounding the disconnect between perceived benefits and adherence to HEPs is that “clear, unified, evidence-based clinical guidelines and recommendations for standard or usual HEPs are lacking” [31].

Moderator Frank DeRuyter introduced the session by first noting that rehabilitation HEP adherence is distinct from treatment adherence (taking medications, etc.). He offered a general definition for the former: the number of exercises documented as completed divided by number of exercises prescribed (A = C/P), a seemingly straightforward definition, but with variables that are quite complex with considerable subtlety. Research conducted by the mRehab RERC shows that clinicians do not always intend all prescribed exercises to be completed, or for all exercise sessions to be completed. In some cases, the prescribed number of sessions and repetitions might be intended as aspirational, in the hope that patients at least do something. Furthermore, measuring or tracking exercise completions is challenging. Patient self-reports are unreliable, while instrumenting patients with trackers introduces complexity, which may serve as an additional barrier to exercise completion.

Dr. DeRuyter reported results from his review of the literature that showed accelerating growth in the number of published peer reviewed articles on patient adherence to treatment (e.g., taking prescribed medications, attending scheduled clinic visits, etc.) and HEPs, especially over the past 2 decades. The volume of research on treatment adherence is much greater than for adherence to HEPs. In the recent decade (2013–2022), there were 156,509 articles on treatment adherence versus 5268 related to HEP. Notably, the volume of research on treatment adherence peaked just before the onset of the COVID-19 pandemic, while scholarship on HEP adherence continued to grow through 2022. Despite this growing research activity, dosage, prescription guidelines and adherence are still not well understood. Although the evidence is mixed [32,33,34], it is generally assumed that non-adherence (non-performance of home exercises) leads to poor outcomes, extended duration of treatment, lower quality of life, increased economic burden, and other negative outcomes. But many studies show adherence levels ranging from 50 to 70% for frequency and duration of performance of home exercises [35,36,37]. Furthermore, a systematic review of older individuals showed that the rate of adherence can range between substantially lower and higher depending on the program and the exercises [38]. This can include health condition, the prescribed intervention regimen, or the measurement techniques and tools utilized. In addition, specific patient-related barriers (i.e., pain, post injury depression, social support) may influence adherence [30].

Questions of dosage and clinician strategies and expectations for adherence were explored further in Dr. Raeda Anderson’s presentation of survey research data on rehabilitation clinicians collected in 2022 by the mRehab RERC team at Shepherd Center [39]. The 293 survey respondents included physical therapists (PTs), occupational therapists (OTs), and speech language pathologists (SLPs) working with rehabilitation patients. HEP adherence was assessed from multiple angles to understand patterns of clinician exercise assignment, perceived patient adherence, and expected patient improvement trajectories. Clinician perceptions of facilitators and barriers to adherence commonly experienced by patients, as well as interventions employed by clinicians to promote HEP adherence, were also explored.

Across the three clinician respondent groups (PT, OT, and SLPs), 84.6% prescribed home exercises, prescribing an average of 4.7 exercises each (2.1 exercise sessions for 21.9 min per day on average) at an average of 5 days per week. The high volume of exercises prescribed to individual patients suggests that when a team representing multiple clinical specialties treats an individual patient, each clinician tends to prescribe exercises without coordination with the other clinical professionals on the therapy team. This can result in the prescription of high numbers of exercises and exercise sessions, requiring considerable time devoted to home exercises. A great majority of rehabilitation clinicians (81%) reported expecting completion of between 40% and 79% of prescribed exercises (consistent with previous research for various patient populations), with PTs reporting the highest expectations for home exercise completion.

Respondents also identified the five most likely barriers to low HEP adherence: (1) motivation level; (2) lack of family and social support; (3) cognitive functioning; (4) pain; and (5) medical status and complications. To overcome barriers, respondents reported the following: (1) modifying the type of exercise; (2) discussing barriers with patients; (3) providing equipment; (4) enhancing social support; and (5) providing technology-supported activity tracking. Notably, 68% of respondents said that technology would improve the level of HEP adherence. These results suggest that further research is needed to understand the barriers and facilitators of technology use to support HEP adherence, measure more precisely the impact of home exercise adherence on patient outcomes, and develop predictive modeling to inform home exercise prescription.

A deeper and more nuanced understanding of individual patients and their socio-economic and cultural background is also critical for greater adherence to health interventions and prescriptions, and for greater uptake and more enduring use of technology supports for HEPs. In her talk, Dr. Sutanuka Bhattacharjya noted that mHealth technology allows patients to engage in in-home rehabilitative activities while monitoring their own performance and managing their own health behaviors [40,41,42]. Furthermore, research has shown a “link between cultural factors and the acceptance of technology” [43]. “Therefore, understanding patient self-management and cultural value systems is an important factor for designing effective self-management interventions that could ultimately influence self-management behaviours” [42]. Indeed, in many cases, the lack of adoption can occur because intervention designers did not consider cultural context when developing these applications [44,45,46,47].

Dr. Bhattacharjya emphasized the need for technology-supported rehabilitation programs to be culturally tailored to increase adoption and ongoing adherence, providing a detailed description of her project to create a nutrition self-management mobile app for low-resource communities in rural India [48,49]. A culturally informed design, she noted, allows the user to successfully perform the task and experience greater satisfaction [50,51,52], which plays a crucial role in the user’s perception of usability [53,54]. Culture affects the preferences of and expectations of users, which include “color, font, images, navigation and the interaction” of the system [54]. Adoption of technology can be influenced by specific cultural norms, beliefs, and behaviors even more so when the technology influences the outcome of one’s health [55,56].

The final presentation in the first session, given by Dr. Candice Osborne, at Craig Hospital, focused on chronic disease self-management solutions, particularly self-management mobile apps. Dr. Osborne noted that many mRehab interventions are designed to support self-monitoring and maintenance of health. These self-management interventions, usually initiated in a clinical setting and intended for long-term adherence in community settings, often include evidence-based education, support for self-care tasks, tracking of biometrics and symptoms, facilitation of provider–patient communication, and support for comprehensive self-management. Almost 7 in 10 adults in the United States (69%) track at least one health indicator using a mobile device, and individuals with chronic conditions [57] are significantly more likely to track health indicators compared to those without chronic conditions [58]. Despite these high rates of self-tracking of personal health indicators, long-term patient adherence to prescribed exercises requires technology that meets the needs and expectations of target users [59]. User-centered content, design, and features are critical to successful adoption and longer-term use of mRehab solutions [60].

### 3.2. Session 2: Technology for Remote Monitoring and Support

The presentations in Session 2 examined a series of technologies developed to support mRehab for people with disabilities, in particular people living with the effects of stroke or traumatic brain injury (Table 2).

The first presentation, given by Dr. Daniel Zondervan, described gamified exercise systems developed by Flint Rehab, LLC, and examined use data to understand patterns of persistence in people with stroke who used the Flint Rehab exercise systems for rebuilding physical function in the home and community. Three of Flint Rehab’s hardware-based systems were described: (1) Music Glove, a single “glove” with conductive fingertips used to play a simple rhythm-based musical game to motivate practice of thousands of gripping movements; (2) FitMi, which uses a “virtual gym” approach to guide users through 40 different exercises performed by moving sensorized “pucks” that detect touches, squeezes, twists, and turns; and ADL Plus, an integrated set of sensorized knobs, switches, door handles, and other everyday tactile interfaces packaged in a portable “suitcase”. All these hardware solutions include electronic games that provide real-time feedback to users. Using sensor technology to support gamification of home exercises can enhance patient engagement in their HEPs, which in turn can lead to improved performance of prescribed exercises and ultimately improved outcomes [61,62].

Dr. Amanda Rabinowitz followed with the presentation of a framework for developing an AI-enabled chatbot to support behavior change interventions, which have been used to support diverse populations, including those with HIV/AIDS, dementia, nutrition and weight loss, mental health, and more [63]. Her team developed RehaBot, an interactive chatbot accessed via text messaging, designed to augment face-to-face behavioral activation (BA) treatment for reducing depression and increasing participation in individuals with moderate to severe traumatic brain injury [64]. Dr. Rabinowitz’s RehaBot intervention is informed by the COM-B model of behavior change [65], which focuses on three core patient characteristics: Capability and Opportunity, which influence Motivation, all of which affect Behavior and patient performance of activities in the home and community. Dr. Rabinowitz also presented the user-centered design process used for developing RehaBot, which is critical to ensuring effective implementation of the core elements of the COM-B model to promote behavior change. Findings from this research provide preliminary evidence suggesting that RehaBot is usable and may promote better adherence to planned target activities [66].

The final presentation in the second session was given by Dr. Marika Demers from the Université de Montreal, who focused on gathering feedback from key stakeholder groups in the user-centered design process for wearable sensors for stroke rehabilitation: patients (people living with stroke), clinicians, and researchers and other experts. Overall, stroke survivors indicated enthusiasm for using wearable technology to support their home-based rehabilitation, and they were able to offer helpful feedback on their motivations and goals, the usefulness of real-time feedback, and the need for continuing engagement with clinicians for exercise prescriptions and to ensure accountability [67]. Clinicians supported the use of wearable sensors to collect objective measures of real-life activity and functioning of their community-dwelling patients. They also valued the data for guiding clinical decisions, supporting patient self-management and engagement, and promoting energy management and personalization. Challenges and concerns were also identified, particularly by R&D experts, who indicated concern about whether existing technologies are adequately mature, technological complexity, privacy and security, adoption by clinicians, and reimbursement by payers.

### 3.3. Session 3: Analytic Techniques for Managing “Big Data” for mRehab

The third session focused on data capture and analytic techniques to support clinical decision making and personalization of rehabilitation interventions and prescription (Table 3). Which data are meaningful? How do you measure activity and physiological status? How do you transform those data into actionable insights to prompt greater adherence and determine the need for progression in a patient’s HEP?

Dr. James Rehg reviewed wearable sensor technologies to capture data that measure target health indicators to support just-in-time adaptive interventions (JITAIs, a key feature of precision rehabilitation). Dr. Rehg’s research on smoking cessation strategies centers on analysis of sensor-generated data to identify stressors (risk markers) that increase vulnerabilities for individuals who may revert to smoking to relieve stress. Effective JITAIs require data on (1) risk markers to identify the appropriate moments for intervention and (2) event detectors to identify and measure risk markers and smoking behavior. The key to JITAI success is context sensitivity, which makes data collection and labeling particularly challenging.

Several challenges must be addressed for the development and implementation of JITAIs: (1) measurement of key causal variables, (2) missing data due to inherent complexity of tracking individuals in the home and community, and (3) lack of sufficient labeled data to train machine learning models. Measuring key variables that are otherwise difficult to capture might be addressed by using indirect or proxy measures (e.g., using the SeismoPatch fabricated at Northwestern University (Evanston, IL, USA) to measure accelerations at the sternum to assess heart function) [68]. “Missingness” in data streams can result from a variety of causes, like sensor placement on the body, battery life, connectivity, and throttling of data transmission speeds by service providers [69]. JITAIs consequently require sophisticated approaches to imputing missing data. Imputation of missing data and data labeling, particularly for data generated by individuals in the home and community, can be achieved through a cross-modal “self-supervised learning” approach that integrates knowledge from a teacher (labeled patterns of data with some features identified of the intended object of measurement), knowledge from the environment (unlabeled patterns with additional features), and knowledge from internal data model activation (self-labeled patterns) [70].

Dr. Keith Lohse followed with a discussion of the need to collect data on the right measures for the right populations. Mobile sensors generate a surplus of data, but measuring meaningful clinical biological, physical, and functional state or activity data in the context of the intended physical activity represents a substantial challenge for researchers and engineers. The need to ensure collection of the right data in context is highlighted by the distinction between capacity (assessed under standardized conditions) for a movement or activity and performance of an activity (what a person actually does in their usual environment) [71,72,73].

Generally, in clinical settings, therapists will measure capacity indicators like range of motion and walking speed. Progress on these indicators can indicate overall functional status but does not indicate what and how much of an activity an individual is performing at home [74,75]. In order to give clinicians actionable feedback to improve care, measurement of activity performance outside the clinic requires a three-step process: (1) verification (precise and unbiased measurement of the property of interest), (2) analytical validation (reliably measuring the intended physiological and behavioral metrics, across a range of input/environmental conditions); and (3) clinical validation (is the technology measuring a meaningful clinical, biological, physical, and functional state or experience in the context of intended physical activity?) [76]. This is a complex process. Despite a surplus of candidate measures, very few have the validation needed for clinical implementation or to be used as outcomes in clinical trials.

Dr. Sangjoon Kim’s presentation focused on the Smart Coach development project that incorporates AI chat routines with sensorized devices and exergames [77] to support adaptive interventions for encouraging stroke patients to engage in therapeutic exercises in the home. Based on prior work on habit formation [78,79,80] and perseverance [81] in performing exercise and other health and wellness programs, Smart Coach utilizes gameplayer data to track engagement and guide the timing and content of messaging designed to encourage engagement over time. From data on game playing on Flint Rehab’s FitMi sensorized devices obtained from over 2000 at-home users, the development team has identified several key factors that can predict perseverance: (1) initial weekly exercise frequency, (2) schedule consistency, (3) exercise repetition rate, and (4) initial success rate (not too hard or easy).

### 3.4. Session 4: Barriers and Facilitators to Uptake and Adoption of mRehab

The final session of the 2-day conference was on barriers and facilitators to mRehab adoption, with particular focus on clinician experience, regulatory environment, and reimbursement and implementation of these technology-enabled interventions on the clinic/institutional level (Table 4).

Dr. Lauri Bishop provided a clinician’s perspective on barriers and facilitators to using and prescribing technology to patients. After summarizing technologies commonly used for upper and lower extremity stroke rehabilitation, Dr. Bishop sorted these solutions into five categories: EMG/FES orthoses, gaming/training devices, exoskeletons, activity monitors, and mobile applications (the last being purely software). She described the main barriers and facilitators to adoption of each category. Her recommendations to therapists considering these technologies include the following: cost and availability, insurance reimbursement, size and weight of the technology, difficulty of donning and doffing, difficulty/ease and time required to configure for different patients, suitability for patients with specific functional limitations, and enjoyment/engagement potential.

Dr. Stephanie Barnes followed with an overview of the regulatory environment and the evolution of federal reimbursement policies for “virtual care management” services in the United States. The Center for Medicare and Medicaid Services (CMS) has led the evolution of public policy in this area, beginning at least as far back as 2013 with new regulations on Transitional Care Management. There have been almost annual new rules and regulations since then, leading to the creation of billing codes for remote physiologic monitoring (RPM) in 2019 and remote therapeutic monitoring (RTM) in 2022. Though these billing codes have been welcomed by healthcare professionals and care delivery organizations, their complexity and sometimes narrow specification has impeded their full adoption and use. The RPM billing codes, for instance, offer reimbursement ranging from USD 19.32 to USD 54.22 (in 2023) and cover different aspects of care management, including initial setup and patient education of equipment, monitoring of physiologic parameters (e.g., weight, blood pressure) by physicians or clinical staff, interactive communication with patients, and transmission of daily recordings. Adoption of the recent RTM codes, which are more relevant to activities associated with managing patient completion of HEPs, has been slow in part due to low reimbursement rates, required minimum number of days of data transmission (16 days within a 30-day period), and lack of awareness among providers of rehabilitation services.

Drs. Veronica Swanson and Mike Jones concluded the session with a summary of their efforts to evaluate introduction of the refined Sensor-Enhanced Activity Management platform (SEAM 2.0) into practice at two clinical settings, outpatient rehabilitation during the pandemic [82] and post-acute rehabilitation setting post-pandemic. The SEAM 2.0 therapy management platform incorporates many of the features of the Flint Rehab system into the Pt Pal therapy management platform. The platform enhancements include the following: (1) sensor-enhanced activity documentation using the exercise devices developed by Flint Rehab (FitMi puck) or attachable sensors (FitMi clip) that can be used to capture a wide variety of movements, (2) a streamlined sensor connection process reducing the number of setup steps required by a therapist or patient, (3) addition of a “reviewed-by” feature into Pt Pal to document when and what exercises are reviewed by the therapist with each patient, and (4) a mechanism to limit the number of active exercises available to the patient from a catalog of recommended exercises. In addition, information about prescription practices of therapists and patient exercise adherence will be available in therapy management “dashboards” being designed for clinical managers and individual therapists to monitor performance. Managers can view a dashboard showing how each therapist is performing with respect to number of active exercises prescribed, frequency of review of exercise results with patients, and overall rates of adherence for their patients. Therapists will also have a dashboard view of how each patient is performing, number of prescribed exercises, schedule of review, etc.

The implementation trial reinforced the importance of several factors that must be considered when implementing a new technology solution into clinical practice: (1) plan data analysis from the onset in implementing a home exercise management system to avoid numerous challenges to ensuring data accuracy; (2) engage clinical team members to ensure system use fits with clinical workflow, particularly with respect to integration with existing ICT demands (e.g., use of an electronic medical record); (3) allocate time and resources for technical training and continued support of therapists and patients; (4) establish expectations about patient adherence; and (5) provide easy-to-obtain and actionable feedback about clinicians and patients expected use of the system to manage adherence.

## 4. Discussion

Discovery of research and development priorities in Round 1 took place at the end of the SOS Conference. It involved a dynamic, interactive in-person session with all presenters, mRehab RERC staff and registered conference attendees. Round 1 produced an extensive list of more than 40 priorities for future research, development priorities to advance the mRehab field (Table 5).

The research and development priorities identified in Round 1′s open ideation session were raw and unrefined, typical of the initial round of discovery in Delphi research studies. These priorities were further refined and condensed by all mRehab RERC staff in Round 2 and subsequently by principal investigators and project directors in Round 3. This process resulted in the following research and development priorities:

### 4.1. Future Research Needs

1. Determine the efficacy of features of HEP management systems in improving exercise adherence.
2. Validate that improved HEP adherence improves health and function outcomes.
3. Identify any potential risks of harm resulting from increased adherence/excessive effort, such as physical injury or psychological stress to perform exercises.
4. Demonstrate the value of mRehab technologies to improve access to rehabilitation for underserved populations (e.g., self-directed vs. clinician-directed and insurance-reimbursed rehabilitation).
5. Determine the importance of patient choice of exercises, intensity, and coaching style. Does it improve adherence? Is progress toward rehabilitation goals the same/faster/slower when patient preferences contribute to exercise prescriptions?
6. Determine the current industry use of CMS RTM billing codes and reimbursement success. What are the best practices for incorporating RTM reimbursement into practice?
7. Understand the effectiveness of various models for behavioral coaching to improve adherence, e.g., preferred interaction style (drill sergeant, coach, cheerleader, delegator), behavioral contingencies, method and frequency of feedback on performance.
8. Understand the influence of bio-psycho-social variables (e.g., social determinants of health, contextual and cultural factors) on adherence and benefits derived from improved adherence to HEPs.

### 4.2. Future Development Needs

1. Design HEP systems with easy setup by patients in the home, device/platform agnosticism, and connectivity to cloud.
2. Continue efforts to integrate HEP systems into clinical practice.
  - Avoid duplication of effort to document/review in the app and in the EMR;
  - Close the feedback loop for patients and clinicians to enable easy access to data on exercise progress;
  - Integrate data for reporting, business intelligence, and automation through AI (e.g., to manage exercise progression) with electronic medical records (EMRs).
3. Employ AI chatbots as a universal interface for patients.
4. Gather input from industry, including ICT companies, developers, and engineers.
  - Complement existing consumer and clinician networks maintained by the mRehab RERC with industry partners;
  - Survey the needs of the field;
  - Help is needed to access/understand the interest of consumers and providers in data sharing for data lake analytic research.
5. Expand data sharing consortium beyond current partners in the mRehab RERC.
  - Modeled after “All of Us” and other consortia;
  - Identify common data elements and measurement methods;
  - Contribute data to expand shared mRehab data lake for analytics research.
6. Establish a “toolkit” for clinical practices on how to optimize reimbursement under RTM codes and share this with industry and clinical partners.

Several of the priorities distilled from our consensus-building process focus on building partnerships or consortia and engaging a diverse community of professionals to advance the field. Since its founding in 2020, the mRehab RERC has been staffed with professionals from a variety of fields, including clinical and social-behavioral researchers, engineers, data scientists and statisticians, clinical rehabilitation professionals, entrepreneurs and business owners, and people with disabling conditions and diseases (e.g., stroke, traumatic injury, neurodegenerative diseases).

Partnerships with private companies in the rehabilitation technology sector are particularly critical for supporting creative thinking and building capacity based on synergies in perspectives and competencies [83,84,85]. However, such public–private or industry–university partnerships can experience some challenges that need to be carefully addressed in organizational design and operations, including weak governance and uneven communication and coordination [83,86] divergent goals among partners [87], management of intellectual property [88,89], and possible power imbalances between partners [89].

Several RERC staff members have majority or minority ownership of mobile rehabilitation technology companies, including Daniel Zondervan, PhD. (President of Flint Rehab), David Reinkensmeyer, PhD (minority owner of Flint Rehab), and John Dzivak and Naveen Khan (co-owners of Pt Pal). The expertise of industry partners in developing and commercializing rehabilitation technology and long-term commitment to their products are critical to ongoing R&D efforts [86,90]. Any concerns about possible research bias or divergent goals can be and have been mitigated by strong governance and open communication among the several members and partner institutions in the mRehab RERC. Future R&D collaborations and partnerships in the field of mobile rehabilitation between academic institutions, private sector companies, and other professional partners “must be developed from trust, legitimacy, and the required capacity” [91].

### 4.3. Limitations

This report and the process for identifying and refining future research and development priorities for mobile rehabilitation have several potential limitations. First, conference organizers diverged from the classic Delphi method in three important ways. In particular, (1) many of the participants in the first round of discovery were not specifically recruited, (2) we allowed face-to-face interaction among the experts in all three rounds of data collection and refinement, and (3) we did not use a formal rating system to evaluate potential priorities. Our approach was adapted to fit the structure and context of holding our conference within a larger annual conference of a professional research society. Consequently, many of the participants in the first round were attendees who chose to register for our conference. The SOS Conference organizers did not specifically invite these attendees or screen them. The conference format also meant that interaction was necessarily face-to-face for Round 1. We chose to continue that face-to-face approach (i.e., no anonymity) for Rounds 2 and 3. Despite this, all participants engaged actively and contributed comments and recommendations for future work that were informed by their direct experience in the field of technology-supported rehabilitation. Finally, we did not use a formal rating system to score the individual recommendations in each round. Again, our approach was adapted to fit the structure and context of our SOS Conference. The direct interaction among experts in all three rounds resulted in dynamic discussion with no apparent inhibition by participants.

The Delphi method in general has limitations, mainly centered around possible bias in the selection of participants and uncertainty regarding participants’ dedication to engaging the topics under discussion [84]. Additionally, interpreting results, even when a formal rating system is used, often relies on the knowledge and judgment of the organizers [92,93].

## 5. Conclusions

The need for continuing research and development of mRehab tools and techniques, along with deeper understanding of paths to successful implementation and reimbursement, will continue to grow as the U.S. and global population continues to age and the gap in supply and demand for health and rehabilitation professionals grows over the next decade. The technological infrastructure (e.g., high speed information and communication networks, ubiquity of powerful sensorized devices like smartphones and wearable sensors, cloud computing, artificial intelligence and machine learning) is in place. Public policy and social attitudes (especially since the start of the COVID-19 pandemic) are increasingly aligned to support expansion of mRehab interventions. Still, challenges remain that will require a massive and sustained effort to design and implement successful mRehab strategies and solutions.

The mRehab RERC State of the Science Conference provided a mechanism to assess the current status and possible future directions for research and development (engineering) in the area of mobile rehabilitation. Utilizing a multi-stage process informed by the Delphi method for expert consensus-building to identify possible future directions allowed mRehab RERC staff to engage scholars and stakeholders to identify emerging trends in healthcare, physical medicine and rehabilitation, technology, society, and public policy. The breadth and dynamism of the field of mobile health and mobile rehabilitation will continue to require ongoing engagement and evaluation of R&D priorities among diverse professionals.

## Figures and Tables

**Table 1:** Session 1: Adherence to and effectiveness of home/remote therapeutic exercise.

Session	Presentation Title	Presenters
1.1	Clinician Strategies and Perspectives on Patient Adherence to Home Exercise Programs	Raeda Anderson, PhD Shepherd Center
1.2	Inclusiveness and Cultural Relevance for Developing mRehab Interventions	Sutanuka Bhattacharjya, OTR/L, PhD Georgia State University
1.3	Patient-Centered mRehab for Self-Management of Chronic Conditions	Candice Osborne, OTR, MPH, PhD, Craig Hospital

**Table 2:** Session 2: Technology for remote monitoring and support.

Session	Presentation Title	Presenters
2.1	Motivating and Monitoring Patient Activity in the Home and Community Using Gamified Sensor Technology	Daniel Zondervan, PhD, Flint Rehab
2.2	Designing and Implementing an AI Conversational Agent for Behavior Activation in People with Brain Injury	Amanda Rabinowitz, PhD, Jefferson Moss Rehabilitation Research Institute
2.3	Perspectives of Stakeholders to Facilitate Uptake and Adoption of Wearable Technology in Stroke Rehabilitation	Marika Demers, PhD, Université de Montréal

**Table 3:** Session 3: Analytic techniques for managing “Big Data” for mRehab.

Session	Presentation Title	Presenters
3.1	Novel Use of mHealth Data to Identify Vulnerability and Receptivity to Just-in-Time Adaptive Interventions	James Rehg, PhD University of Illinois Urbana Champaign
3.2	Translating Accelerations into Participation: Big Data, Latent Variables, and Challenges of Actigraphy	Keith Lohse, PhD Washington University
3.3	Building Automated Chatbot Coaching System to Encourage Effective Engagement with a Home Rehabilitation Game for Stroke Survivors	Sangjoon Kim, PhD University of California Irvine George Collier, PhD Shepherd Center

**Table 4:** Session 4: Barriers and facilitators to uptake and adoption of mRehab.

Session	Presentation Title	Presenters
4.1	Barriers and Facilitators to Integrating Mobile Rehabilitation Technologies into Clinical Practice	Lauri Bishop, DPT, PhD University of Miami
4.2	Regulatory and Reimbursement Environment for mHealth and mRehab Interventions and Future Directions	Stephanie D. Barnes, JD, PhD Nixon Gwilt Law
4.3	Implementation of an mRehab Architecture in Outpatient Clinics	Veronica Swanson, PhD University of California, Irvine Mike Jones, PhD, FACRM Shepherd Center

**Table 5:** Round 1 of expert opinion data gathering: research, development, and training.

Development/Engineering	Research
Research to prove outcomes	Clinical reports to include adherence
What is adherence? Two levels of adherence • Home exercise prescriptions; • Technology adherence (technology use).	Data-capture from multiple providers
	Capture multiple dimensions of activity + context
	Use sensors with other platforms (e.g., SMS)
Use technology to inform dosage	Train sensor during in-person session
Patient choice	Setting thresholds for risk of non-adherence
Patient level data	Timely feedback to patient
For, by, about patients	Universal chatbot for multiple devices
Is a snapshot model good enough?	AI-informed program for customization
Identify risk of harm from HEPs	Use sensors for hard-to-measure activity and status
• Does tech impact risk?	Data labeling research
Identify minimum threshold for improvement	Tag motion for accuracy
Design mechanisms for ensuring data quality	Micro RCTs to refine coaching
AI/ML/chat routines	Capacity building—create post doc positions
Compare therapist + patient perspectives	Toolkit to get RPM adopted
Capture patient context	Technology and industry network
Content of coaching	Foundation for data sharing
Incorporate patient choice	Gather evidence
Build data ocean (versus “data lake”)	Crowdsourced Wiki for rehab tech solutions
Data sharing by companies and researchers	• Independent confirmation of posts by SMEs
Community context	• Curation site for research and data
Include social determinants of health (SDOH)	• Group training + active training
Environmental factors: weather, day, time, place	

## Data Availability

No new data were created or analyzed for this report. Data sharing is not applicable to this article.
